# Artificial intelligence to enhance research integrity in evidence-based medicine: toward scalable trustworthiness assessment

**DOI:** 10.3389/frma.2026.1833223

**Published:** 2026-07-08

**Authors:** Moses Mo, Rohit Arora, Christian Cao, Andrea C. Tricco, David Moher

**Affiliations:** 1 Cumming School of Medicine, University of Calgary, Calgary, AB, Canada; 2 Harvard University, Cambridge, MA, United States; 3 University of Toronto, Toronto, ON, Canada; 4 Unity Health Toronto, Toronto, ON, Canada; 5 Li Ka Shing Knowledge Institute, St. Michael's Hospital, Toronto, ON, Canada; 6 Centre for Implementation Research, Ottawa Hospital Research Institute, Ottawa, ON, Canada; 7 School of Epidemiology and Public Health, University of Ottawa, Ottawa, ON, Canada; 8 Dalla Lana School of Public Health, University of Toronto, Toronto, ON, Canada

**Keywords:** artificial intelligence, evidence synthesis, integrity analytics, machine learning, research integrity, retractions, systematic reviews, trustworthiness assessment

## Abstract

Fraudulent, falsified, and otherwise unreliable clinical research threatens the foundations of evidence-based medicine because systematic reviews, meta-analyses, and clinical practice guidelines depend on the trustworthiness of the studies they include. Retractions are increasing faster than publication output and often occur only after substantial delay, allowing problematic trials to be cited, pooled, and incorporated into downstream clinical recommendations. Recent evidence shows that retracted randomized trials have contaminated thousands of meta-analyses and hundreds of guideline documents, while only a small minority of affected reviews later self-correct. This is not only a clinical problem but also an integrity analytics problem: unreliable studies generate article-level, trial-level, and network-level signals that are not yet systematically integrated into evidence-synthesis workflows. Existing approaches can detect some image anomalies, textual irregularities, reporting inconsistencies, and statistical red flags, but important blind spots remain. Fabricated clinical datasets may appear statistically plausible, outcome switching often requires registration-publication comparison, and most available tools operate in isolation rather than as coordinated screening systems. We argue that the next generation of safeguards should combine AI/ML-based detection systems, statistical forensic methods, structured trustworthiness appraisal tools, and workflow-level screening frameworks within evidence synthesis. Embedding staged trustworthiness assessment early in systematic reviews, alongside stronger publisher- and database-level integrity infrastructure, could help prevent unreliable trials from distorting pooled estimates and downstream guidance. Such systems should support triage and prioritization rather than replace human judgment. Framed in this way, scalable trustworthiness assessment represents a practical research metrics and analytics agenda for strengthening the evidence ecosystem from primary studies to reviews, guidelines, and policy decisions.

## 1 Introduction

Fabrication, falsification, and plagiarism (FFP) are problematic behaviors in the production of science. Fraudulent or otherwise unreliable evidence from clinical trials undermines evidence-based medicine because systematic reviews and clinical practice guidelines depend on the integrity of the studies they include. When fabricated or later-retracted studies are incorporated into meta-analyses, their misleading results can propagate into misleading clinical recommendations that can directly shape patient care (Xu et al., 2025). The Retraction Watch Database now lists more than 55,000 retractions, including over 10,000 issued in 2023 alone (Staff, 2024; Van Noorden, 2023b). Although detection tools have advanced, critical blind spots remain, particularly in clinical trials and systematic reviews, which enable unreliable data to persist within the evidence ecosystem (Xu et al., 2025; Carlisle, 2021; Wilkinson et al., 2025).

## 2 Retractions are rising faster than publication output

The scale of research misconduct is accelerating. Between 2000 and 2022, the compound annual growth rate of retracted papers was 22.09%, far exceeding the 6.25% growth rate of total publications (Zhou et al., 2025). The average retraction rate across disciplines from 2000 to 2024 was approximately 11 per 10,000 papers, though rates vary widely by field. Among top-cited scientists, clinical medicine and biomedical research have the highest retraction prevalence, with 3.3–4% of top-cited authors in these fields having at least one retraction (Ioannidis et al., 2025b). Subfields spanning the translational-clinical spectrum, including complementary and alternative medicine (10.5%), oncology (9.9%), and pharmacology (9.4%), are particularly affected, while physics and mathematics have near-zero rates.

Most retractions stem from misconduct rather than honest error: Fang and colleagues found that 67.4% of retractions were attributable to misconduct, including fraud (43.4%), duplicate publication (14.2%), and plagiarism (9.8%), while only 21.3% reflected honest mistakes (Fang et al., 2012). More recently, large-scale paper mill activity—fraudulent organizations that produce and sell fabricated manuscripts or authorship slots—has emerged as a dominant driver, with estimates suggesting that as many as 400,000 papers published over the past two decades exhibit strong textual similarities to known paper mill products (Van Noorden, 2023a; Parker et al., 2024).

Further compounding the problem, retractions occur slowly: the mean time from publication to retraction is approximately 34 months, and for papers authored by serial offenders (“super-retractors”), time to retraction may exceed a decade (Steen et al., 2013; Lyu et al., 2025). During this window, flawed studies can be cited, meta-analyzed, and embedded into clinical guidelines. Detection itself remains largely reactive: volunteer “data sleuths,” working outside formal institutional structures, have become an effective mechanism for uncovering research misconduct. Bik and colleagues manually screened over 20,000 biomedical papers and found inappropriate image duplication in ~4%, with at least half of the problematic cases suggestive of deliberate manipulation (Bik et al., 2016). In a separate journal-level analysis, identified duplications prompted dozens of editorial actions, including 41 corrections and 5 retractions (Bik et al., 2018).

That said, systems and infrastructure to detect retractions have improved. Post-publication scrutiny increasingly occurs on PubPeer, which has triggered high-profile institutional investigations (Caron et al., 2025). The Retraction Watch Database, acquired by Crossref in 2023, is now accessible via API and downloadable datasets (Retraction Watch, 2026; Oransky, 2023). Additional tools such as the STM Integrity Hub and various publisher-specific AI systems screen manuscripts pre-publication (STM Association, 2026). Yet these systems primarily target obvious red flags such as text duplication, image manipulation, and citation anomalies, rather than deeper threats to study trustworthiness.

## 3 Retractions propagate into systematic reviews, with consequences

The 2025 VITALITY study identified 1,330 retracted randomized controlled trials contaminating 4,095 meta-analyses from 847 systematic reviews (Xu et al., 2025). Retracted trials changed the direction of pooled effects in 8.4% of meta-analyses and statistical significance in 16.0% of contaminated meta-analyses. Evidence from 68 distorted systematic reviews fed into 157 clinical practice guideline documents. Despite this, only 5% of systematic reviews actually corrected themselves following retraction (Avenell et al., 2024). The implication is clear: retraction alone does not protect the evidence ecosystem. Without automated detection and correction mechanisms, fraudulent trials can remain embedded in the secondary literature long after their removal from the primary record. Viewed through this lens, contamination of evidence syntheses is not merely a downstream consequence of delayed retraction; it is a measurable propagation failure that highlights where workflow-integrated trustworthiness assessment could intervene before unreliable trials distort secondary analyses and guideline development.

## 4 Several detection tools have emerged across biomedical publishing

AI-powered tools promise to scan the literature for red flags at an unprecedented scale. In image analysis, computer-vision systems can now compare figures across millions of papers. Services like ImageTwin and Proofig use machine learning to spot identical or manipulated images like flipped, rotated, or spliced Western blots across publications (Imagetwin, 2026; Proofig AI, 2026; Chaturvedi et al., 2025). Publisher-developed tools add additional layers: Springer Nature's Geppetto detects AI-generated nonsense text (a classic paper mill indicator), while SnappShot analyzes gel and blot images for duplications (Springer Nature, 2026; Springer Nature Group, 2026). On the statistical front, tools like Statcheck automatically scan papers' reported p-values and test statistics, recomputing them to catch inconsistencies that might indicate misreporting (Nuijten and Polanin, 2020). These developments represent early but promising steps toward more scalable, AI-assisted quality control in scholarly publishing, including plagiarism and similarity screening and the detection of near-duplicate or simultaneously submitted manuscripts. Representative tools and their corresponding integrity signals, lifecycle stages, and blind spots are summarized in Table 1.

**Table 1:** Representative tools for detecting unreliable research, grouped by category.

Category	Representative tools	Signal detected	Stage in lifecycle	Key blind spots
Image analysis (computer vision/ML)	ImageTwin, Proofig, SnappShot	Duplicated, spliced, or manipulated figures (e.g. Western blots, gels)	Pre- and post-publication manuscript screening	Fabricated numerical or patient-level data; novel AI-generated images
Text and language screening (NLP)	Geppetto, similarity/plagiarism checkers	AI-generated or “tortured” text; duplicate or near-duplicate submissions	Pre-publication submission screening	Fluent, plausibly written fabricated text from current LLMs
Statistical consistency and forensics	Statcheck, GRIM, SPRITE, Carlisle method	Internal statistical inconsistency; implausible summary statistics or baseline distributions	Post-publication or summary-statistic review	Correctly computed fabricated data; single-center trials without cross-site comparison
Trial-data distribution monitoring (AI/ML)	CluePoints SMART	Anomalous data patterns across trial sites	Active trial conduct/monitoring	Not designed for retrospective screening of published literature; single-site trials
Structured trustworthiness appraisal (rule-/checklist-based)	INSPECT-SR, RIA	Composite trustworthiness (registration, ethics, reporting, plausibility)	During systematic review, before data extraction	Labor-intensive; relies on human application; misses statistically plausible fabrication on its own
Registration cross-referencing	COMPare (manual); proposed automation	Outcome switching; protocol–publication discrepancies	Eligibility/full-text assessment	Currently manual; not yet automated at scale
Network and systemic indicators	Ioannidis ranking, RI2, predatory-journal watchlists, Retraction Watch DB, PubPeer	Author-, institution-, and venue-level risk; retraction status	Macro-level triage/prioritization	Probabilistic; risk of unfair attribution and reputational harm
Reporting-guideline adherence (LLM)	AutoReporter	Item-level adherence to reporting guidelines	Post-publication/screening	Adherence to reporting standards is not equivalent to truthfulness

For each category the table lists representative tools, the type of integrity signal targeted, the stage of the publication and evidence-synthesis lifecycle at which the tool is most applicable, and its principal blind spots.

## 5 The detection gap

Despite these advances, clinical data FFP, and synthetic datasets remain largely hidden to algorithmic detection. Fabricated patient-level data can be statistically plausible and lack the obvious anomalies that would trigger automated flags. A 2021 audit by editors of the journal *Anaesthesia* analyzed individual participant data from 153 clinical trials submitted between 2017 and 2020, finding that 44% contained false data and 26% contained fabricated data (Carlisle, 2021).

Clinical trial data manipulation takes forms distinct from basic science fraud. The most common methods include outright data fabrication, data falsification, selective reporting, outcome switching, and post hoc hypothesis or outcome modification. The COMPare Trials Project, examining 67 trials in five top medical journals, found that each trial reported only 25–96% of pre-specified primary outcomes while silently adding an average of 5.4 new outcomes per trial (Goldacre et al., 2019).

Several forensic mechanisms exist. The GRIM test checks whether reported means are mathematically possible given sample sizes (Brown and Heathers, 2017). The SPRITE method reconstructs plausible datasets from summary statistics (Nielsen et al., 2025; Heathers et al., 2018). The Carlisle method evaluates whether baseline-variable distributions are consistent with genuine randomization; in an analysis of 5,087 RCTs, a stringent probability threshold flagged ~1.6% of unretracted trials as having highly improbable baseline patterns and, using an author-level heuristic, highlighted Boldt, Fujii, and Reuben, three authors with multiple retracted trials (Mascha et al., 2017; Carlisle, 2017). One advanced AI/ML application is the CluePoints SMART platform, which uses unsupervised statistical models to compare data distributions across trial sites; the FDA signed a 3-year agreement with CluePoints in 2024 to develop AI algorithms for clinical trial oversight (Sagefrog, 2024; Cluepoints, 2026). The INSPECT-SR tool, developed with Cochrane backing and endorsed in October 2025, provides 21 trustworthiness checks for assessing RCT reliability during systematic review (Wilkinson et al., 2025). Unlike conventional risk-of-bias tools, INSPECT-SR targets signals of potentially problematic trials (including critical error or possible fabrication) that can slip into meta-analyses and guidelines. Cochrane recommends using it early, before data extraction, to avoid investing effort in trials that may ultimately be deemed unreliable.

Despite this growing toolkit, critical blind spots persist. If a fabricator generates plausible patient data and calculates statistics correctly, GRIM, SPRITE, and Statcheck will not detect it. Single-center trials lack the cross-site comparisons that monitoring tools depend on. No integrated screening pipeline exists: current tools operate in isolation, with no unified system combining statistical, textual, image, and network analyses. Meanwhile, generative AI can now produce convincingly fabricated clinical papers, raising the prospect of synthetic trials that evade traditional red flags entirely.

## 6 Where do we go from here

An integrated clinical trial integrity platform should combine automated statistical forensics with image integrity screening, NLP-based language analysis, cross-referencing of trial registrations with publications, and social network analysis of authors and institutions. No widely adopted integrated system exists today. The CluePoints-FDA collaboration represents the most advanced step toward this goal, but it focuses on monitoring active trials rather than screening the published literature retrospectively.

Automated cross-referencing of ClinicalTrials.gov registrations with published results represents a major unexploited opportunity. The COMPare project demonstrated that outcome switching is pervasive but currently trackable only through laborious manual comparison (Goldacre et al., 2019). Machine learning systems could systematically compare registered protocols, including primary and secondary endpoints, sample sizes, and timelines, with published reports to detect outcome switching and selective reporting at scale.

Such a platform could be implemented as a staged workflow within evidence synthesis. At study identification, automated checks could screen for retraction status, trial registration, duplicate publication, and basic text or image anomalies. During full-text eligibility assessment, protocol-publication cross-referencing could compare prespecified outcomes, sample sizes, timelines, and ethics approvals against the published report, while author- and institution-level metadata could be used to flag higher-risk studies. Before data extraction and risk-of-bias assessment, structured trustworthiness tools such as INSPECT-SR could then be applied preferentially to flagged trials, with statistical forensics and targeted image review reserved for cases in which concerns persist. Final inclusion or exclusion decisions would allow AI to function as a triage and prioritization layer rather than an autonomous adjudicator. The proposed layered workflow for integrating article-level, trial-level, and network-level signals into evidence synthesis is summarized in Table 2.

**Table 2:** A layered framework for workflow-integrated trustworthiness assessment.

Layer	Signals	Methods/tools	Point of application	Output/action
Article-level	Text, image, and statistical anomalies	NLP screening; image forensics; Statcheck, GRIM, SPRITE	Study identification/screening	Flag study for closer inspection
Trial-level	Registration–publication discrepancies, outcome switching, reporting and ethics inconsistencies, implausible baseline data	Registration cross-referencing; Carlisle method; INSPECT-SR, RIA	Full-text eligibility, before data extraction	Triage; preferential structured trustworthiness appraisal
Network-level	Author, institution, and venue risk patterns; retraction histories; predatory-journal indicators	Institutional ranking, RI2; Retraction Watch DB; watchlists	Prioritization across the candidate set	Risk score to prioritize human review
Workflow actions	(Decisions informed by the layers above)	Human-in-the-loop adjudication	Inclusion/exclusion and correction propagation	Include, exclude, escalate, or trigger correction of affected reviews and guidelines

Integrity signals are organized into three levels: article-level (text, image, and statistical anomalies), trial-level (registration–publication discrepancies, outcome switching, and implausible baseline data), and network-level (author-, institution-, and venue-based risk patterns).

Perhaps the most actionable near-term advance lies in embedding research integrity assessment directly into the systematic review process. A recent Cochrane ivermectin review offers a compelling case study: its authors developed a Research Integrity Assessment (RIA) tool, a hierarchical, six-domain framework applied during eligibility screening, before risk-of-bias assessment (Popp et al., 2022; Weibel et al., 2023). The RIA evaluated retraction status, prospective trial registration, ethics approval, author group plausibility, reporting adequacy, and result plausibility (Weibel et al., 2023). Application of the RIA led to the exclusion of more than 40% of candidate trials in the first update, materially shifting the results and conclusions (Weibel et al., 2023). In the resulting Cochrane synthesis after RIA screening, ivermectin showed no clear mortality benefit compared to standard of care or placebo, with confidence intervals crossing 1 (Popp et al., 2022). This demonstrates how inclusion of FFP trials can distort pooled estimates in meta-analysis.

At the systemic level, emerging tools aim to identify where problematic research may be concentrated. Ioannidis and colleagues recently proposed an institutional ranking system that adjusts raw research impact for three integrity-related penalties: retraction authorships, excessive self-citation rates, and publication in discontinued (often predatory) journals (Ioannidis et al., 2025a). Applied to nearly 7,000 institutions, the ranking revealed stark geographic patterns, with integrity penalties disproportionately concentrated in specific countries and institutions. The parallel Research Integrity Risk Index uses similar signal categories (Meho, 2025). These macro-level indicators, combined with predatory journal watchlists, can guide where resources for integrity vetting are most needed.

Machine learning could further scale these efforts. Classifiers trained on features of known problematic trials, such as implausible effect sizes, inconsistent baseline characteristics, absent trial registration, or affiliation with flagged institutions, could assign risk scores to trials entering systematic reviews. Such a system would not replace human judgment but could prioritize which studies warrant closer inspection, making integrity vetting feasible even for large-scale evidence syntheses.

AutoReporter, an LLM-based tool that automates item-level assessment of research reporting guideline adherence at scale, demonstrates that structured AI-driven screening of biomedical literature is possible (Chen et al., 2026). The same underlying capabilities could be extended to automate INSPECT-SR-type trustworthiness checks, systematically screening for statistical anomalies, inconsistencies in reported methods, and implausible participant data across large volumes of trials simultaneously. When further layered with cross-referenced metadata on predatory journal classifications, suspected paper mill authorship networks, and individual author retraction histories, such a system would offer a composite integrity risk profile that moves fraud detection from a reactive, post-publication exercise to a scalable gatekeeping layer embedded prospectively within the evidence synthesis pipeline: a function no existing tool currently achieves at scale.

Automated integrity screening introduces risks that must be managed if these systems are to support rather than damage the evidence ecosystem. First, no detector is error-free; incorrectly flagging a sound trial can delay legitimate evidence, consume scarce reviewer effort, and damage reputations, and the probabilistic author- and institution-level signals we describe are especially prone to unfair attribution. Such signals must never function as automated verdicts. Second, these methods are vulnerable to adaptation; as detection of tortured phrasing, image duplication, and statistical inconsistency becomes routine, sophisticated fabricators can engineer outputs that pass each individual check, so detection and evasion co-evolve and any single-layer system degrades over time. Third, screening carries real computational and financial costs, and the licensed tools, compute, and expertise it requires are unevenly distributed. Without deliberate design, integrity screening could entrench rather than reduce existing inequities, disadvantaging reviewers, journals, and research communities in low- and middle-income settings, the same communities on which macro-level integrity indices already concentrate penalties. Leveling the field therefore depends less on the sophistication of any algorithm than on whether screening capacity is shared as common infrastructure rather than gated behind publisher- or institution-level resources. For these reasons, the systems we propose are best understood as triage and prioritization layers operating under human oversight, with transparent, explainable outputs and documented error rates, rather than as autonomous arbiters of research integrity.

## 7 Closing remarks

A central governance question remains: who should bear responsibility for implementing scalable trustworthiness safeguards? At present, much of the burden falls on systematic review authors, who often lack the tools, training, and institutional support required to conduct rigorous integrity vetting. That arrangement is neither efficient nor sustainable. A more realistic model is one of distributed responsibility across the evidence ecosystem. Bibliographic databases and indexing services should further standardize and expose structured integrity metadata, including retraction status, expressions of concern, duplicate-publication links, and trial-registration linkage where available. Publishers should continue to strengthen pre-publication screening for text, image, and metadata anomalies. Systematic reviewers should apply structured trustworthiness assessment early, before data extraction, particularly for studies that trigger predefined concern signals. Guideline developers and evidence services should also maintain mechanisms for updating recommendations when included reviews are later affected by serious integrity concerns. Under such a shared-responsibility model, trustworthiness assessment becomes distributed rather than an unfunded burden placed almost entirely on review authors. The cost of inaction is measured not only in corrupted literature, but also in the patients, clinicians, and policy actors who rely on evidence that may never have been trustworthy enough to enter the evidence base in the first place.

## Data Availability

The original contributions presented in the study are included in the article/supplementary material, further inquiries can be directed to the corresponding author.
